# Isolation and Characterization of Human Trophoblast Side-Population (SP) Cells in Primary Villous Cytotrophoblasts and HTR-8/SVneo Cell Line

**DOI:** 10.1371/journal.pone.0021990

**Published:** 2011-07-07

**Authors:** Tomoka Takao, Kazuo Asanoma, Kiyoko Kato, Kotaro Fukushima, Ryosuke Tsunematsu, Toshio Hirakawa, Sueo Matsumura, Hiroyuki Seki, Satoru Takeda, Norio Wake

**Affiliations:** 1 Department of Obstetrics and Gynecology, Graduate School of Medical Sciences, Kyushu University, Higashi-ku, Fukuoka, Japan; 2 Department of Obstetrics and Gynecology, Juntendo University, Bunkyo-ku, Tokyo, Japan; 3 Sanada Women's Clinic, Higashi-ku, Fukuoka, Japan; 4 Department of Nutrition Management, Faculty of Health Science, Hyogo University, Kakogawa-shi, Hyogo, Japan; 5 Department of Obstetrics and Gynecology, Saitama Medical Center, Saitama Medical University, Kawagoe-shi, Saitama, Japan; Baylor College of Medicine, United States of America

## Abstract

Recently, numerous studies have identified that immature cell populations including stem cells and progenitor cells can be found among “side-population” (SP) cells. Although SP cells isolated from some adult tissues have been reported elsewhere, isolation and characterization of human trophoblast SP remained to be reported. In this study, HTR-8/SVneo cells and human primary villous cytotrophoblasts (vCTBs) were stained with Hoechst 33342 and SP and non-SP (NSP) fractions were isolated using a cell sorter. A small population of SP cells was identified in HTR-8/SVneo cells and in vCTBs. SP cells expressed several vCTB-specific markers and failed to express syncytiotrophoblast (STB) or extravillous cytotrophopblast (EVT)-specific differentiation markers. SP cells formed colonies and proliferated on mouse embryonic fibroblast (MEF) feeder cells or in MEF conditioned medium supplemented with heparin/FGF2, and they also showed long-term repopulating property. SP cells could differentiate into both STB and EVT cell lineages and expressed several differentiation markers. Microarray analysis revealed that IL7R and IL1R2 were exclusively expressed in SP cells and not in NSP cells. vCTB cells sorted as positive for both IL7R and IL1R2 failed to express trophoblast differentiation markers and spontaneously differentiated into both STB and EVT in basal medium. These features shown by the SP cells suggested that IL7R and IL1R2 are available as markers to detect the SP cells and that vCTB progenitor cells and trophoblast stem cells were involved in the SP cell population.

## Introduction

Human placenta is a unique organ associated with fetomaternal circulation, which involves decidua basalis as the maternal component and chorionic villi from the fetus. Placenta consists of trophoblasts, which exhibit several functions such as protection, nutrition and respiration of fetus, as well as hormone production [1]. Placental trophoblasts include relatively undifferentiated villous cytotrophoblast (vCTB), intermediate cytotrophoblast, terminally differentiated villous syncytiotrophoblast (STB) and extravillous cytotrophoblast (EVT) that invade into maternal decidua. These differentiated trophoblasts arise from a putative trophoblast stem (TS) cell population; it has been proposed that vCTB at the villous basement membrane contains a TS cell population.

In a previous study, mouse TS cell lines were established using blastocysts and extraembryonic ectoderm of E6.5 embryos cultured *in vitro* [2]. They are self-renewable in the presence of FGF4 and feeder cells, and then readily differentiate into diverse trophoblast cell lineages in the absence of FGF4 and feeder cells [2], [3]. Human TS cell lines cannot be established under conditions similar to that used for mouse TS cell lines [4]. The differentiation of human embryonic stem cells into trophoblasts has been studied under certain conditions [5]–[10]. Most of the studies failed to show induction of CDX2, EOMES or ERRB in the trophoblasts, so it is controversial whether they are real human TS cells. Only a few groups presented CDX2 positive cells as putative human TS cell compartments [9], [10].

vCTB contains progenitor cells, and possibly, TS cells that continue to produce daughter cells that differentiate and fuse with syncytium or differentiate into invasive trophoblasts [11]–[13]. vCTB cells can be isolated from human villous tissue at any stage of pregnancy for primary culture [14], [15]. Several groups performed flow cytometric analysis to screen for the expression of markers of primary vCTB cells. However, no studies have successfully identified reliable human TS markers [16]–[18].

Stem cells have been identified in diverse adult tissues and play a critical role in tissue homeostasis throughout life. Somatic stem cells are defined as undifferentiated cells because of their ability to both self-renew and differentiate to produce mature progenitor cells at the single cell level. In 1996, hematopoietic stem cells with immature characteristics were isolated from a specific cell population called side-population (SP) cells [19]. SP cells have the unique ability to pump out DNA binding dye Hoechst 33342 via the breast cancer resistance protein1/ATP-binding cassette (ABC) transporter, subfamily G (BCRP1/ABCG2) [20]. To date, SP cells have been isolated from several normal tissues, including blood [21], intestine [22], liver [23], lung [24], muscle [25], skin [26], uterus [27], [28], testis [29], and mammary gland [30]. The ability of SP cells to rapidly efflux Hoechst 33342 has been used to isolate SP cells by flow cytometry and cell sorting.

In this study, we isolated and analyzed SP cells from a human trophoblast cell line, HTR-8/SVneo and human primary vCTB. By immunocytochemistry and gene expression analysis at the genome-wide level, SP cells were suggested to include vCTB stem cells/ progenitor cells. They showed long-term repopulating capability and differentiated into multiple trophoblast cell lineages *in vitro*. We also identified IL7R and IL1R2 as two excellent markers to separate SP population from non-SP (NSP) population.

## Results

### Isolation and characterization of SP cells from HTR-8/ SVneo cell line

**Figure 1:**
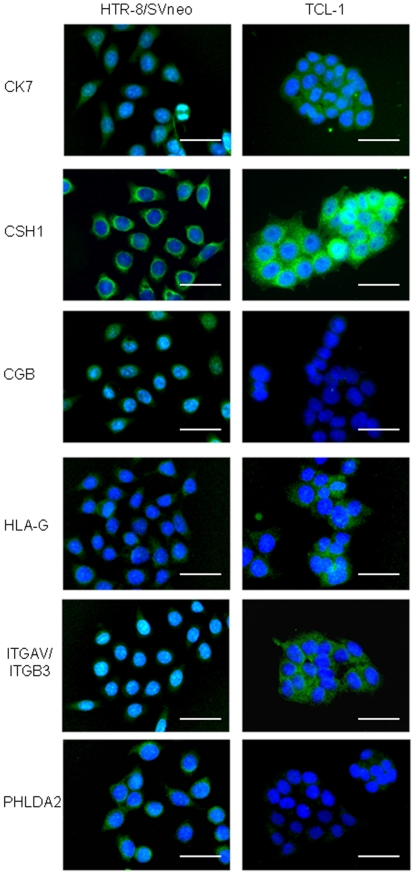
Characterization of HTR-8/SVneo and TCL-1 cell lines. Immunocytochemistry of HTR-8/SVneo and TCL-1 cells with the trophoblast differentiation markers CK7, CSH1, CGB, HLA-G, ITGAV/ITGB3 and PHLDA2 (scale bars, 50 µm). Each protein expression is shown as green signals and nuclei are shown as blue signals. TCL-1 cells showed abundant expression of EVT markers, HLA-G and ITGAV/ITGB3. In contrast, HTR-8/SVneo cells showed abundant expression of CGB and PHLDA2 but low expression of HLA-G and ITGAV/ITGB3.

Many human trophoblast cell lines have been established, which essentially originated from one of two sources: from normal tissues or from malignant tissues. For our purpose of studying villous cytotrophoblast (vCTB) progenitor cells, cancer cell lines were thought to be inappropriate because of their aberrant gene expression that developed in the process of carcinogenesis. Because of the absence of proper vCTB cell lines that precisely reflect vCTB *in vivo*, we first tested an immortalized human trophoblast cell line, HTR-8/SVneo, which is known to have originated from extravillous cytotrophoblast (EVT) [31]. Another trophoblast cell line, TCL1, which has also been used as an EVT cell line, was used for comparison with HTR-8/SVneo. HTR-8/SVneo was established by transfection of human primary trophoblasts, which originated from first trimester villous explants, with a gene encoding simian virus 40 large T antigen to immortalize them [32]. On the other hand, the other cell line, TCL-1, was established by retroviral expression of simian virus 40 large T antigen in primary culture of choriodecidua of a term placenta. A single cell was isolated from it and a clone that could repopulate for long term was established [33]. In TCL-1 cells, immunocytochemistry showed strong expression of HLA–G and ITGAV/ITGB3, which are known as EVT markers [34]–[36]. TCL-1 cells showed an undetectable level of expression of CGB, which is a villous syncytiotrophoblast (STB) marker [37] (Figure 1). TCL-1 cells also showed no expression of PHLDA2, which is an imprinted gene product reported to be strongly expressed in vCTB but absent or expressed only weakly in STB and EVT [38], [39] (Figure 1). In contrast, HTR-8/SVneo cells showed abundant expression of CGB and PHLDA2, but only weak expression of HLA-G and ITGAV/ITGB3 (Figure 1). As expected, both cell lines expressed cytokeratin 7 (CK7), a pan-trophoblast marker, and CSH1, which is expressed in STB and EVT [37], [40], [41] (Figure 1). These data suggested that the nature and origin of HTR-8/SVneo were different from those of TCL-1, which were closer to the EVT signature. Furthermore, hierarchical clustering analysis and principal component analysis based on genome-wide gene expression profiles showed that HTR-8/SVneo had large differences from primary EVT [42]. On the basis of this evidence, we speculated that HTR-8/SVneo was not completely committed to EVT and had several features of villous trophoblast.

**Figure 2:**
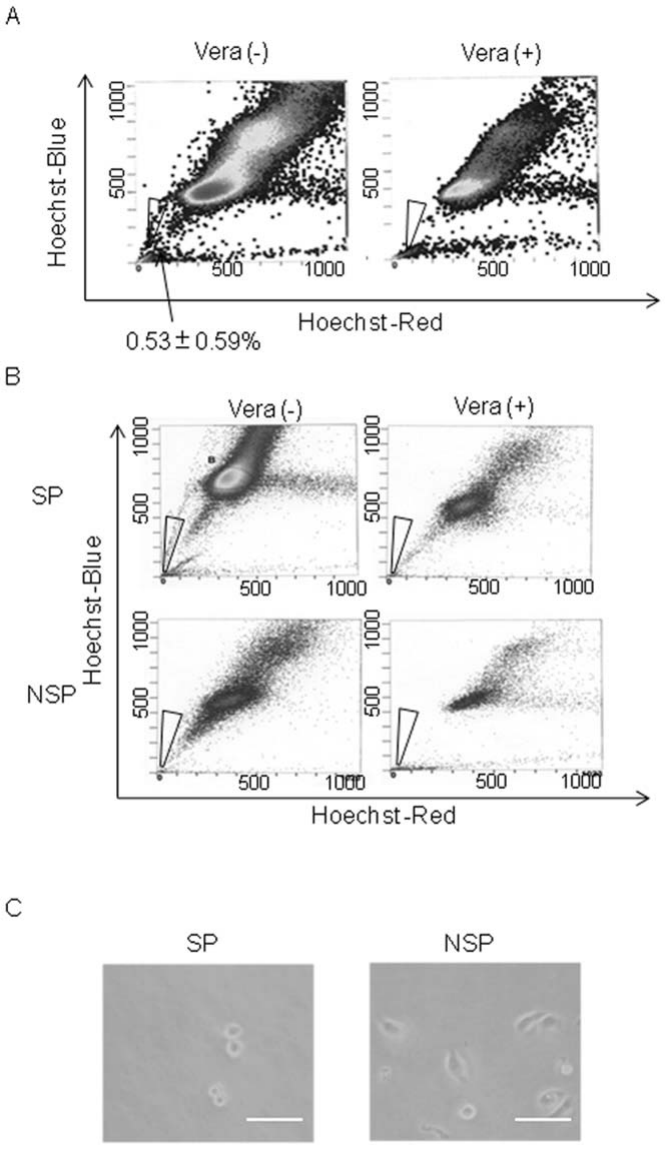
Isolation of SP and NSP cells from HTR-8/SVneo cell line. (A) Flow cytometry results of HTR-8/SVneo cells stained with Hoechst 33342 with or without verapamil (Vera). SP cells were obtained from HTR-8/SVneo cells (0.53±0.59% from 4 independent experiments). Verapamil treatment blocked the dye efflux, increased staining and rendered the SP cells undetectable by flow cytometry. The cell fraction gated with a triangle is SP. (B) Both HTR-8/SVneo-SP cells and -NSP cells were reanalyzed by flow cytometry after culture for 3 weeks in HBM. SP and NSP subpopulations were obtained again from the former HTR-8/SVneo-SP cells. In contrast, NSP cells produced only NSP fraction. Similar results were obtained from three independent experiments. (C) Morphologically, the SP cells were small and round in shape, in contrast to NSP cells. The NSP cells were spindle-shaped (scale bars, 50 µm).

HTR-8/SVneo cells were stained with Hoechst 33342 and sorted by Cell Sorter Epics ALTRA. SP cells were detected at 0.53±0.59% out of total HTR-8/SVneo cells (Figure 2A), and disappeared upon addition of 100 µM verapamil, which is characteristic of SP cells. In contrast, SP cells could not be obtained in TCL-1 cell line (data not shown). These contrasting results supported the hypothesis that the nature and origin of HTR-8/SVneo were different from those of TCL-1, which should be committed to EVT and does not include progenitor cells. Next, both HTR-8/SVneo-SP cells and -non-SP (NSP) cells were cultured for 3 weeks in HTR-8/SVneo basal medium (HBM), stained again with Hoechst 33342 and analyzed using the cell sorter. SP and NSP fractions were obtained again from the former SP subpopulation after being cultured for 3 weeks. In contrast, SP fraction could not be obtained from the former NSP population (Figure 2B). Morphologically, SP cells were small, round and showed a large nucleus-to-cytoplasm ratio (Figure 2C), but NSP cells were spindle-shaped and elongated. Given the fact that mouse TS cells are cuboidal or round and have a large nucleus-to-cytoplasm ratio, HTR-8/SVneo-SP cells were morphologically similar to mouse TS cells.

**Figure 3:**
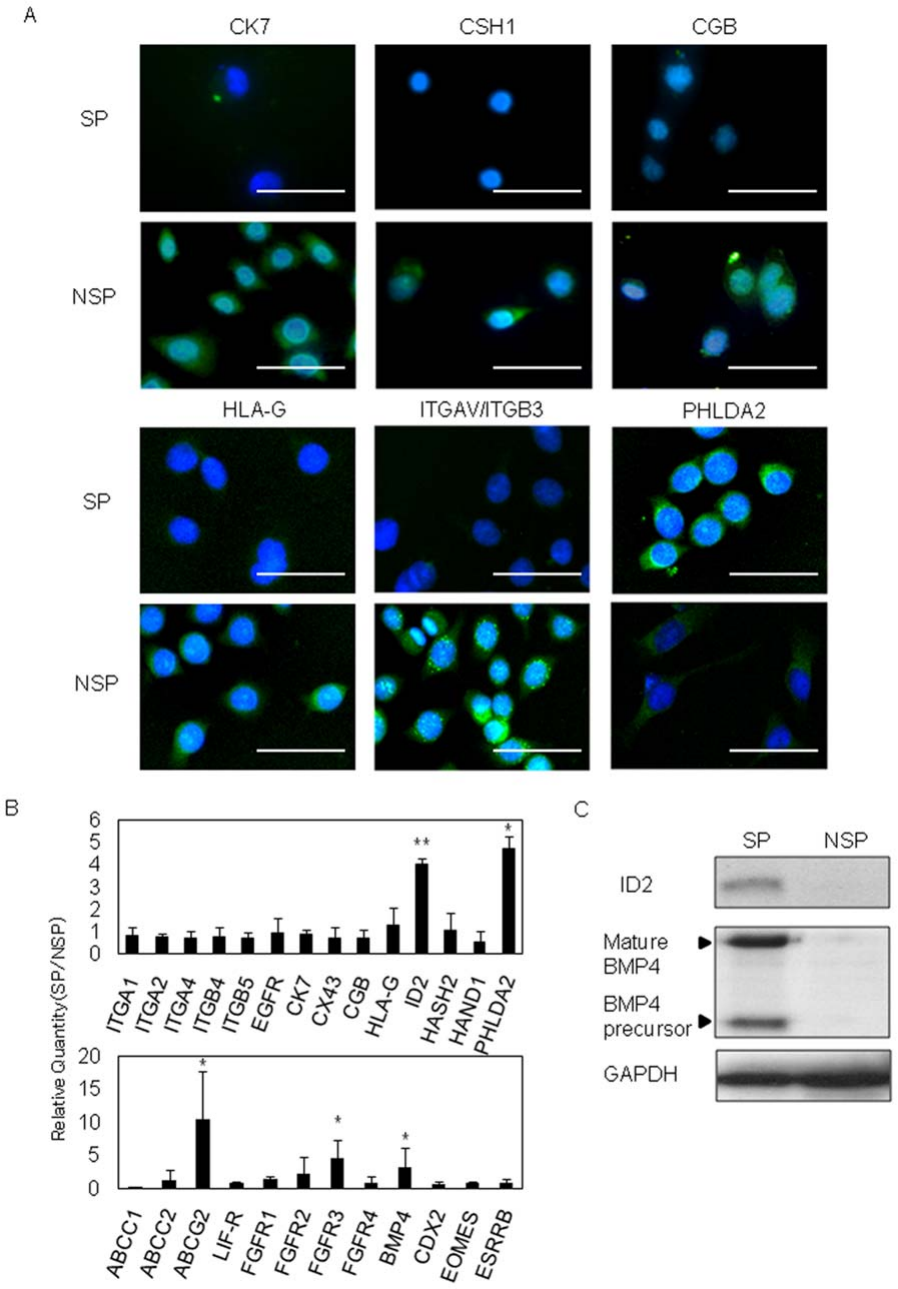
Characterization of SP and NSP cells from HTR-8/SVneo cells. (A) Immunocytochemistry with the trophoblast differentiation markers, CK7, CSH1, CGB, HLA–G and ITGAV/ITGB3, and PHLDA2, on HTR-8/SVneo-SP and -NSP cells (scale bars, 50 µm). Each protein expression is shown as green signals. (B) Comparison of gene expression between HTR-8/SVneo-SP and -NSP cells by real-time RT-PCR analysis. Fold changes of mRNA level in SP cells against NSP cells on each transcript are shown. The messenger RNA levels were normalized to that of GAPDH and error bars show standard deviations (n = 3). Asterisks indicate statistically significant (*, *p<*0.05; **, *p<*0.01). (C) Western blot analysis revealed that HTR-8/SVneo-SP cells expressed ID2 and BMP4 (both mature and precursor) proteins. In contrast, NSP cells failed to express them.

A variety of marker expression was compared between SP and NSP cells by immunocytochemistry, real-time RT-PCR and Western blotting. Among various markers examined, mRNA expression of PHLDA2, ABCG2, inhibitors of DNA binding protein 2 (ID2), bone morphogenetic protein 4 (BMP4) and FGFR3 was statistically different in SP from that in NSP cells (Figure 3B, *p<*0.05). Most of the isolated HTR-8/SVneo-SP cells expressed undetectable level of CSH1, CGB, HLA-G and ITGAV/ITGB3. In contrast, NSP cells showed abundant expression of these trophoblast differentiation markers (Figure 3A). Although CK7 is recognized as a pan-trophoblast marker, CK7 protein was expressed only in NSP cells. SP cells showed 4.75±0.55 folds higher PHLDA2 mRNA expression compared to NSP (Figures 3B, *p<*0.05). To support this, PHLDA2 protein was exclusively expressed in SP cells (Figure 3A). Compared with NSP, SP cells also showed 4.02±0.29-fold higher mRNA level of ID2, which was reported to be a marker of vCTB stem cells [43] (Figures 3B, *p<*0.01). ID2 protein was detectable exclusively in SP cells (Figure 3C). BMP4 is known to induce human embryonic stem cell differentiation into trophoblasts [5]. BMP4 mRNA level was 3.20±2.57 folds higher in SP than NSP cells (Figure 3B, *p<*0.05). Exclusive expression of BMP4 protein was observed in SP cells (Figure 3C). One of the ATP binding cassette (ABC) transporters, ABCG2, has a close relationship with SP cells, having been described by its ability to efflux Hoechst 33342 [44]. As expected, 10.50±7.13 folds higher ABCG2 mRNA expression was observed in SP cells than in NSP cells (Figure 3B, *p<*0.05).

### SP cells could sustain SP characteristics on mouse embryonic fibroblast (MEF) feeder cells or in conditioned medium from MEF

**Figure 4:**
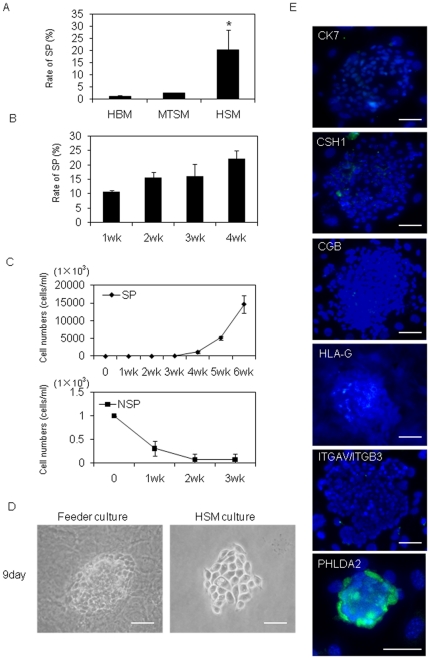
SP cells had capacity for long-term proliferation and self-renewal. (A) SP cells derived from HTR-8/SVneo were cultured in HBM, MTSM and HSM for 3 weeks. SP fraction was analyzed by Epics ALTRA (n = 3). Asterisk indicates statistically significant (*p<*0.05). (B) HTR-8/SVneo-SP cells cultured in HSM were analyzed by Epics ALTRA every week through 4 weeks (n = 3). (C) Cell numbers of HTR-8/SVneo-SP and -NSP cells cultured in HSM were counted for 6 weeks. After 4 weeks, SP cells began to grow, while NSP cells stopped growing after 3 weeks. Data of cell numbers are represented as the mean ± SEM from three independent experiments. (D) Morphologically, the colonies of SP cells cultured in HSM were similar to those cultured on feeder cells. (E) Images of HTR-8/SVneo SP cell colonies cultured on feeder cells immunostained with the trophoblast differentiation markers, CK7, CSH1, CGB, ITGAV/ITGB3, HLA-G and PHLDA2 counterstained with Hoechst 33852. Each protein expression is shown as green signals (scale bars, 50 µm).

SP cells were isolated from HTR-8/SVneo cells at extremely low incidence. In the case of human first trimester villous tissues, several factors have been reported to promote cell proliferation [45]–[47]. To enrich the SP cell fraction, we examined 5 different growth factors in HTR-SVneo basal medium (HBM) (Table1). TGFA, which is expressed in all trophoblast lineages [48], TGFB, which inhibits cytotrophoblast (CTB) proliferation [49], EGF, which is expressed in villous trophoblast lineages [48], IGF2, which enhances CTB proliferation [50] or basic FGF (FGF2), which is expressed in CTB [51] were examined on SP cells. Among these 5 factors, FGF2 gave the best SP recovery rate (data not shown). 25 ng/ml FGF2 was supplemented with SP medium thereafter. To estimate the receptor for FGF2, relative mRNA level between SP and NSP cells (SP/NSP) was examined on each of FGF receptor 1– 4. FGFR3 expression was 4.62±2.74 folds higher in SP cells, that is highest among other members (Figure 3B, *p<*0.05). For mouse TS cells, the standard medium has been reported to contain a mixture of 70% MEF conditioned medium with 30% Mouse TS basal medium. We tested mixtures with several different ratios of MEF conditioned medium and HTR-8/SVneo basal medium (HBM) for optimal HTR-8/SVneo SP medium formation. As a result, 50% HBM with 50% MEF conditioned medium gave the best SP recovery rate (data not shown). This medium was defined as HTR-8/SVneo SP medium (HSM) (Table 1). SP cells were also examined in mouse TS stem cell medium (MTSM), HSM and HBM for four weeks and stained with Hoechst 33342 followed by analyses using Epics ALTRA. Recovery rates of SP cell fraction isolated from the SP cells cultured in these different media were 2.36±0.37% (MTSM), 20.55±11.37% (HSM) and 1.09±0.54% (HBM) (Figure 4A).

**Table 1:** List of culture media.

Medium	Components
HTR-8/SVneo basal medium (HBM)	RPMI1640 supplemented
	10 %FCS
	1 % penicillin and streptomycin
HTR-8/SVneo SP medium (HSM)	50 % HBM+ 50%MEFs conditioned medium produced in HBM supplemented with
	25 ng/ml FGF2
	1 µg/ml heparin
Mouse TS basal medium (MTBM)	RPMI1640 supplemented
	20%FCS
	100 µM 2-mercaptoethanol
	2 mM L-glutamine
	1 % penicillin and streptomycin
Mouse TS stem cell medium (MTSM)	30 % MTBM+ 70% MEFs conditioned medium produced in MTBM supplemented with
	25 ng/ml FGF4
	1 µg/ml heparin

The growth properties of SP and NSP cells cultured in HSM were analyzed for 6 weeks. Recovery rates of SP fraction from the SP cells in HSM remained relatively high even after culture for 4 weeks (Figure 4B). Both SP and NSP cells grew slowly for the first 3 weeks of culture. However, SP cells started to grew prominently after culture for four weeks (Figure 4C). In contrast, NSP cells stopped growing after 4 weeks of culture. We cultured about 1000 single SP cells in HSM or in HBM with MEF feeder cells on 10 cm^2^ dishes. SP cells formed colonies on both of the media conditions, while NSP cells could not. Morphology of SP cell colonies cultured in HSM was similar to that cultured in HBM with MEF feeder cells (Figure 4D). These results suggested that SP cells maintain the capacity of long-term proliferation. Next, we investigated whether these colonies from SP cells expressed trophoblast differentiation markers such as CK7, CSH1, CGB HLA-G or ITGAV/ITGB3 by immunocytochemistry after being cultured for 9 days in HBM with MEF feeder cells. The expression of the differentiation markers was extremely low in the SP cells compared with that of NSP cells, which is contrast to the exclusive expression of PHLDA2 in SP cells (Figures 4E).

### SP cells strongly expressed IL7R and IL1R2

**Figure 5:**
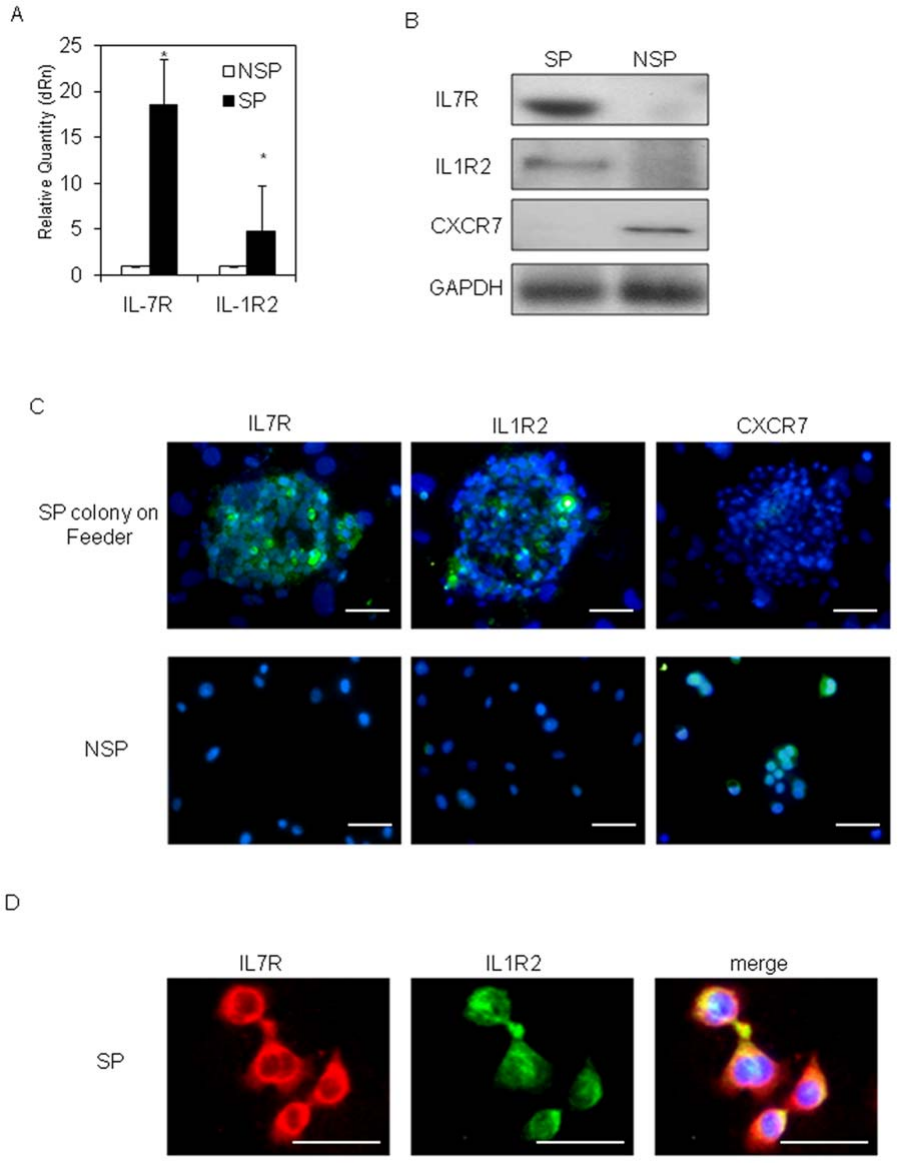
IL7R and IL1R2 were abundantly expressed in HTR-8/SVneo-SP cells. (A) IL7R and IL1R2 mRNA expressions between HTR-8/SVneo-SP and -NSP cells were compared by real-time RT-PCR. The mRNA levels were normalized to those of GAPDH and error bars show standard deviations (n = 3). Asterisk indicates statistically significant (*p<*0.01). (B) Western blot analysis of IL7R, IL1R2 and CXCR7 proteins in HTR-8/SVneo-SP and -NSP cells. SP cells exclusively expressed IL7R and IL1R2, while NSP exclusively expressed CXCR7. (C) The protein expression of IL7R, IL1R2 or CXCR7 was analyzed by immunocytochemistry. All images counterstained with Hoechst 33852. Each protein expression is shown as green signals (scale bars, 50 µm). (D) IL7R and IL1R2 expressions in HTR-8/SVneo-SP cells were analyzed by immunocytochemistry. All images were counterstained with Hoechst 33852. IL7R and IL1R2 expressions are shown as red and green signals respectively (scale bars, 50 µm).

To search for genes that were expressed specifically in SP or in NSP cells, we extracted total RNA from SP cells or NSP cells of HTR-8/SVneo cells and performed DNA microarray analysis using the HG-U133 Plus 2.0 Affymetrix GeneChips. The entire microarray data were deposited in Gene Expression Omnibus (GEO) under accession number GSE27909 (http://www.ncbi.nlm.nih.gov/geo/query/acc.cgi?acc=GSE27909). We identified 155 genes that were upregulated in SP cells more than three-fold higher than that in NSP cells. Additionally, the expression of 986 genes were downregulated in SP less than 0.33 fold compared with those in NSP (refer to GEO: http://www.ncbi.nlm.nih.gov/geo/query/acc.cgi?acc=GSE27909). Each of Table 2 and Table 3 lists the top 20 genes in terms of either upregulation or downregulation, respectively. Among the genes listed, we focused on two cell membrane surface receptors, interleukin 7 receptor (IL7R, also known as CD127) and interleukin 1R type 2 (IL1R2, also known as CD121b). These two genes showed the highest SP/NSP expression ratios among the genes listed (Table 2). Expression of the genes in SP and NSP cells was validated by real-time RT-PCR (Figure 5A) and Western blot (Figure 5B). Immunocytochemistry using the antibodies against these gene products was performed on colonies of SP cells and on NSP cells (Figure 5C). SP cell colonies were strongly positive for IL7R and IL1R2 expression and negative for CXCR7 expression (Figure 5C). In contrast, NSP cells were negative for IL7R and IL1R2 expression and positive for CXCR7 expression. CXCR7 expression was reported in differentiated trophoblasts of term placentas [52]. Most of SP cells were positive for both IL7R and IL1R expression (85.29±4.72%, n = 4) (Figure 5D).

**Table 2:** Representative genes, whose expressions were upregulated in HTR-8/SVneo-SP, compared to –NSP cells.

Gene symbol	Gene title	HTR8-SP/NSP Signal log ratio
IL7R	interleukin 7 receptor	6.6
AURKB	aurora kinase B	6.2
KRTAP3-1	keratin associated protein 3-1	6.1
DNAJC15	DnaJ (Hsp40) homolog, subfamily C, member 15	6.1
THADA	thyroid adenoma associated	6
GTSE1	G-2 and S-phase expressed 1	5.3
IL1R2	interleukin 1 receptor, type II	5.2
SPANXB1/2	SPANX family, member B1/2	5.2
MUC7	mucin 7, secreted	5.1
KMO	kynurenine 3-monooxygenase	4.8
CTGF	connective tissue growth factor	4.5
INTS4	integrator complex subunit 4	4.5
ATAD3A/B	ATPase family, AAA domain containing 3A/B	4.4
GAS2L3	growth arrest-specific 2 like 3	4.3
RAB3B	RAB3B, member RAS oncogene family	4.3
GTSE1	G-2 and S-phase expressed 1	4.2
PDGFRL	platelet-derived growth factor receptor-like	4.2
SERPINB2	serpin peptidase inhibitor, clade B, member 2	4.2
NEXN	nexilin (F actin binding protein)	4.1
CAPSL	calcyphosine-like	4.1

**Table 3:** Representative genes, whose expressions were downregulated in HTR-8/SVneo-SP, compared to –NSP cells.

Gene symbol	Gene title	HTR8-SP/NSP Signal log ratio
NUPR1	nuclear protein 1	−6.4
CLGN	calmegin	−5.4
GPNMB	glycoprotein (transmembrane) nmb	−4.8
BEX2	brain expressed X-linked 2	−4.2
MAF	v-maf musculoaponeurotic fibrosarcoma oncogene homolog	−4.1
AKR1C1	aldo-keto reductase family 1, member C1	−4
KLHDC1	kelch domain containing 1	−3.9
ABCA1	ATP-binding cassette, sub-family A, member 1	−3.8
CG012	hypothetical gene CG012	−3.8
TSC22D3	TSC22 domain family, member 3	−3.8
ECM2	extracellular matrix protein 2	−3.7
TRIM22	tripartite motif-containing 22	−3.7
MAP1B	microtubule-associated protein 1B	−3.7
ZNF750	zinc finger protein 750	−3.6
ABCG1	ATP-binding cassette, sub-family G, member 1	−3.6
RP11-298P3.3	CG016	−3.6
LOC153222	adult retina protein	−3.5
ARG2	arginase, type II	−3.5
C4orf34	chromosome 4 open reading frame 34	−3.5
TNFSF10	tumor necrosis factor superfamily, member 10	−3.5

**Figure 6:**
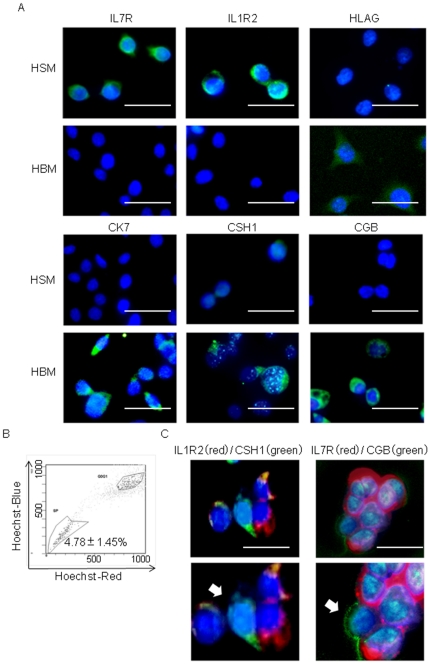
SP and IL1R2/IL7R double-positive cells differentiated into multiple trophoblast cell lineages. (A) Immunocytochemistry images of IL7R, IL1R2, CK7, CSH1, CGB and HLA−G expression in SP cells sorted from HTR-8/SVneo parent cells. Each protein expression is shown as green signals (scale bars, 50 µm). The cells were assayed after culture for 2 weeks in HSM or HBM. (B) Flow cytometry results of IL1R2-positive cells after being sorted by MACS. SP cells were obtained from IL1R2-positive cells after culture for 2 weeks (4.78±1.45% from 3 independent experiments). (C) Induction of trophoblast differentiation of IL7R/IL1R2 double-positive HTR-8/SVneo cells confirmed by differentiation marker, CSH1 and CGB expression. IL7R and IL1R2 expressions are shown as red signals; CSH1 and CGB expressions are shown as green signals (scale bars, 50 µm). The cells marked with an arrow show positive for CSH1 or CGB and negative for IL1R2 or IL7R expression.

Next, to investigate whether differentiation of SP cells was affected by culture conditions, we examined SP cells cultured in HSM or HBM for two weeks. SP cells cultured in HSM showed low expression of trophoblast differentiation markers and high expression of IL7R and IL1R2 (Table 4, Figure 6A). However, culture of SP cells in HBM resulted in an increase in the number of cells expressing trophoblast differentiation markers (CK7, CSH1, CGB and HLA−G) and a decrease in the number of cells with IL7R and IL1R2 expression (Table 4, Figure 6A). These results again suggested that HSM was a more suitable medium for SP cells to sustain the SP characteristics and HBM induced spontaneous differentiation in SP cells.

**Table 4:** Percentages of HTR-8/SVneo-SP cells positive for each marker expression after 2 weeks culture in HSM or HBM.

	Percentages of positive cells (%)		
Gene symbol	HSM	HBM	*p*
CK7	11.87±3.99	35.23±9.85	<0.05
CSH1	14.29±9.85	32.29±1.29	<0.05
CGB	5.24±0.27	35.40±6.33	<0.05
HLA-G	11.78±6.47	47.69±14.3	<0.01
IL7R	88.80±5.83	20.72±3.08	<0.01
IL1R2	63.04±7.85	34.85±1.97	<0.05

HTR-8/SVneo-SP cells were cultured in HSM or HBM for two weeks and examined by immunocytochemistry. Positive cells in terms of expression of each marker were counted under a fluorescence microscope. Data are from three independent experiments. 300 cells were examined for each experiment.

To further examine the impact of IL1R2 expression in SP cells, we isolated IL1R2-positive cells by magnetic cell sorting (MACS). After MACS, the cells were cultured in HSM for 3 weeks and SP cells were isolated by Epics ALTRA. SP cell populations in IL1R2-positive cells were significantly enriched compared with those from HTR-8/SVneo parent cells (Figures 2A and 6B). Because most IL1R2-positive SP cells simultaneously expressed IL7R (Figure 5D), we examined IL7R/IL1R2 double-positive cells thereafter. To characterize the in vitro differentiation ability of the IL7R/IL1R2 double-positive cells, we cultured these cells in HBM for 2 weeks and assessed the expression of trophoblast differentiation markers by immunocytochemistry (Figure 6C). After 2 weeks of culture in HBM, the IL7R/IL1R2 double-positive cells gave rise to a renewed mixture of two cell subpopulations, which were positive for either SP markers (IL7R, IL1R2) or differentiation markers (CGB, CSH1) (Figure 6C). These data suggested that some of the cell population sorted as IL7R/IL1R2 double-positive turned negative for IL7R and IL1R2, but positive for CGB and/or CSH1 as a result of spontaneous differentiation.

Our detailed analysis on HTR-8/SVneo-SP cells suggested that SP cells IL7R/IL1R2 double-positive cells had the characteristics of vCTB progenitor and could differentiate into STB and EVT, which implied that HTR-8/SVneo was not fully committed to EVT and included a cell population with a multi-lineage differentiation capability.

### Isolation of SP and IL7R/IL1R2 double-positive cells in human villi

**Figure 7:**
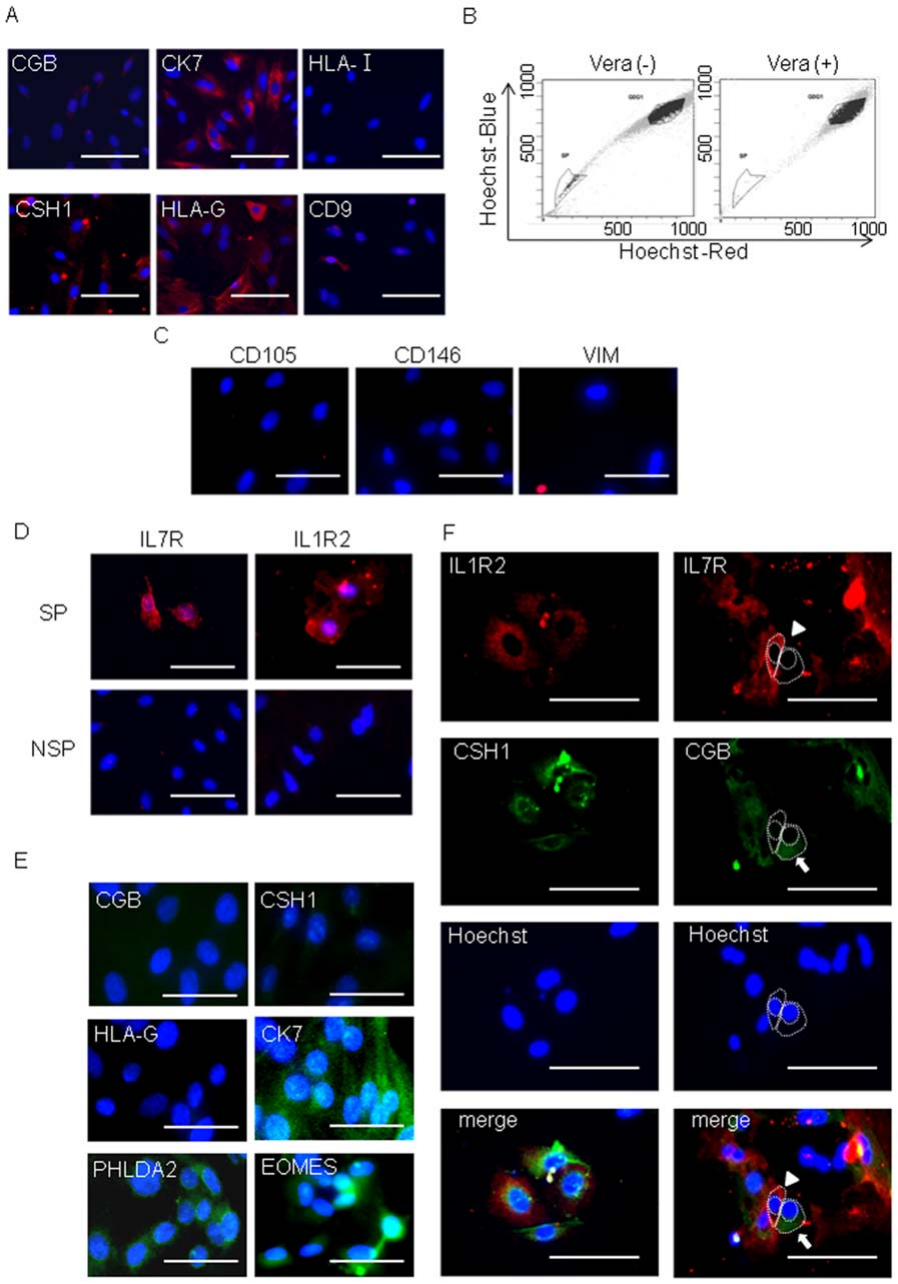
Isolation of SP cells from human first trimester vCTB. (A) Immunocytochemistry images of CGB, CK7, CSH1, HLA−G, CD9 and HLA1 expression in primary vCTB cells isolated by Percoll gradient centrifugation. Each protein expression is shown as red signals (scale bars, 50 µm). The isolated cells were plated on chamber slides with HBM and stained the next day. (B) The SP cells represent 0.12±0.28% (n = 22) of the total primary vCTB. Co-incubation of the cells with verapamil erased the SP cell fraction. (C) The expression of CD105, CD146 and VIM in SP cells from primary vCTB cells was analyzed by immunocytochemistry. All images show counterstaining with Hoechst 33852. Each protein expression is shown as red signals (scale bars, 50 µm). (D) Immunocytochemistry images of IL7R and IL1R2 expression in SP and NSP cells counterstained with Hoechst 33852. IL7R and IL1R2 expressions are shown as red signals (scale bars, 50 µm). (E) The expression of CGB, CSH1 HLA−G, CK7, PHLDA2 and EOMES in IL7R/IL1R2 double-positive cells was assessed by immunocytochemistry. These expressions are shown as green signals (scale bars, 50 µm). (F) Induction of differentiation in IL7R/IL1R2 positive cells was determined by IL7R, IL1R2, CSH1 and CGB expression. IL7R/IL1R2 positive cells from primary human vCTB were analyzed after being cultured in HBM for 2 weeks. IL7R and IL1R2 expressions are shown as red signals; CSH1 and CGB expressions are shown as green signals (scale bars, 50 µm). The cell marked with an arrow show positive for CGB and negative for IL7R expression. In contrast, the cell marked with an arrowhead was positive for IL7R and negative for CGB expression. The outlines of the cells are indicated by broken lines.

To identify SP cells in human villi from 6– 9 gestation weeks, villous tissues were mechanically and enzymatically dissociated and vCTB cells were isolated by percoll gradient centrifugation. These vCTB cells quickly showed spontaneous differentiation into multiple trophoblast cell lineages in HBM. Their differentiation was confirmed by induced expression of CGB, CK7, CSH1 and HLA−G (Figure 7A). HLA class1 and CD9 were reported to be useful to exclude all cells except villous trophoblast [53], [54]. CD9 was also reported to be a specific marker of EVT progenitor cells and its expression was shown to be lost in mature EVT [55]. Purity of the isolated trophoblast was confirmed by the absence of HLA class 1 and CD9 expression (Figure 7A). We obtained an extremely small fraction of SP cells in human villi (0.12±0.28 %, n = 22) (Figure 7B). Contamination of mesenchymal cells and fibroblasts in SP fraction was ruled out by the absence of CD105, CD146 and VIM expression [56]−[59] (Figure 7C). All of the SP cells expressed IL7R and IL1R2, but NSP cells failed to express IL7R and IL1R2 (Figure 7D). Next we isolated IL7R/IL1R2 double-positive cells by MACS. To confirm the immaturity of the double-positive cells, marker expression of the cells was examined after culture in HSM for 2 weeks. The expression of differentiation markers was almost undetectable in IL7R/IL1R2 double-positive cells. In contrast, 51.79±7.49% of the double-positive cells showed marked expression of EOMES, which is a well-known TS cell marker in mice [60] (Figure 7E). The double-positive cells also expressed PHLDA2, an SP-specific marker (Figure 7E). These data suggested that HSM was also an appropriate medium to inhibit differentiation of IL7R/IL1R2 double-positive cells from human villous tissues. Next, the sorted IL7R/IL1R2 double-positive cells were cultured in HBM for 2 weeks to induce differentiation. The cells were positive for either SP markers (IL7R, IL1R2) or differentiation markers (CGB, CSH1) (Figure 7F). These results suggested that IL7R/IL1R2 double-positive cell population in human villi included vCTB progenitor cells with multi-lineage differentiation capability. Most of the SP and IL7R/IL1R2 double-positive cells kept to proliferate and maintained the SP morphology in HSM without differentiation for at least 2 weeks but the cells ceased to proliferate and differentiated soon after that (data not shown). Furthermore, because SP cells and IL7R/IL1R2 double-positive cells isolated from human villi were too few in number, the growth property and long-term repopulation of the cells could not be assessed.

## Discussion

It has been proposed that human placenta villi contain a population of stem cells with remarkable regenerative capability. vCTB in particular undergo asymmetric cell division, which gives rise to a progenitor cell that maintains the pool and another cell that differentiates and fuses with syncytium or differentiates into invasive trophoblast [11−13], .

In this study, HTR-8/SVneo was selected to analyze SP cells. Although many trophoblast cell lines have been established, no cell lines that faithfully reflect the features of vCTB are available. HTR-8/SVneo was generated from primary villous explants of early pregnancy. This suggests that HTR-8/SVneo is heterogeneous in terms of cell population. Several different trophoblast cell types including villous trophoblast and EVT were expected to be present in the original cell population. In fact, the expression profile of trophoblast differentiation markers demonstrated that HTR-8/SVneo included cells expressing vCTB, STB and EVT markers (Figure 1). Additionally, a genome-wide study revealed that HTR-8/SVneo had large differences in gene expression profile from primary EVT [42]. In contrast, TCL-1 originated from a single cell isolated from primary culture of choriodecidua of a term placenta. Therefore, TCL-1 cells should be homogeneous as a cell group. In our results, TCL-1 showed clear characteristics as EVT cells and no SP cells could be obtained from TCL-1. In turn, SP cells could be obtained from HTR-8/SVneo, and they exhibited features of stem cells/ progenitor cells, which include differentiation into multiple trophoblast cell lineages, colony formation, long-term proliferation and self-renewal (Figure 4). Numbers of adult stem cell/ progenitor cell subpopulations have been defined using the fluorescent dye Hoechst 33342 in various human tissues [62]. ABCG2 expression was described to efflux Hoechst 33342 from SP cells [44]. High expression of ABCG2 was also observed in HTR-8/SVneo-SP cells (Figure 3B). Furthermore, our SP cells exclusively expressed ID2, which was reported to be a marker of vCTB stem cells [43] (Figures 3B and C). These data suggested that our trophoblast SP fraction included a stem cell/progenitor cell population. Markedly abundant expression of BMP4 was observed in SP but not in NSP cells (Figures 3B and C). BMP4 is known to induce human embryonic stem cell differentiation into trophoblast [5]. There are no studies about BMP4 expression in placenta. Considering previous reports, our results suggested that BMP4 secreted by trophoblast stem cells/progenitor cells could be important for trophoblast commitment at an early stage or for maintenance of trophoblast cell lineages. We also demonstrated that SP cells expressed some trophoblast differentiation markers, CSH1, CGB and HLA−G as a result of spontaneous differentiation in HBM (Table 4, Figure 6). CK7 has been recognized as a key marker identifying all different trophoblast subtypes. Surprisingly, SP cells and IL7R/IL1R2 double-positive cells from both HTR-8/SVneo and primary vCTB cells expressed an undetectable level of CK7. NSP cells or spontaneously differentiated cells from SP or IL7R/IL1R2 double-positive cells gained abundant CK7 expression (Figures 3A and 6A). Our result may be compatible with a previous report that CK7 was absent in HTR-8/SVneo cells and was re-expressed by converting the culture condition [63]. Some groups that successfully obtained cytotrophoblast progenitor cells from human embryonic stem cells could not demonstrate upregulation of CK7 in the progenitor cells [5], [9]. The work of these groups and our data suggest that CK7 expression was upregulated in the process of trophoblast differentiation from the progenitor cell stage. In murine placenta, Cdx2, Eomes and Errb are known to be critical for the maintenance of TS cells. In human, CDX2 was reported to be differentially expressed in trophectoderm and not in inner cell mass [64]. Furthermore, villous tissues from human early pregnancy were reported to show a higher level of CDX2 expression than those from term placentas [10]. On the other hand, although many trials have been undertaken to obtain human TS cells from human ES cells, most of the studies failed to show induction of CDX2, EOMES or ERRB in their cells [5]−[10]. Although EOMES expression was observed in IL7R/IL1R2 double-positive cells from primary trophoblast culture, HTR-8/SVneo SP cells did not show upregulation of CDX2, EOMES or ERRB compared with NSP cells (Figures 3B and 7E). Careful examination is needed to elucidate the requirements of CDX2, EOMES or ERRB expression for maintenance of human trophoblast stem cells.

In addition to HTR-8/SVneo cells, some of these results were also confirmed using human primary vCTB. We could also obtain SP cells and IL7R/IL1R2 double-positive cells from human primary vCTB and found that they expressed EOMES, a TS marker. The cells also remained immature in HSM medium and could differentiate into multiple trophoblast cell lineages including STB and EVT (Figure 7). Although most of the SP and IL7R/IL1R2 double-positive cells maintained the ability to proliferate and retained the SP morphology in HSM without differentiation for at least 2 weeks, the cells ceased to proliferate and differentiated soon after that (data not shown). This result suggested that the cells require some other factors to maintain their immature properties.

In our study, a number of different culture conditions were tested on SP cells for optimization. SP cells grew better and retained the SP characteristics on MEF feeder cells or in conditioned medium from MEF feeder cells, just like the case with mouse TS cells. Several different growth factors were also tested on SP cells. Because FGF2 gave the best result, FGF2 was chosen for a supplement with our SP medium, HSM. Although vCTB cells can be isolated from villi at any stage of pregnancy for primary culture, they quickly cease proliferating and differentiate within about 5 days [14], [15]. Previous studies reported that proliferation in 1^st^ trimester villous explant could be increased at 4–5 weeks by supplying EGF [45] or IGF [46]. FGF4 was also reported to inhibit differentiation of the 1^st^ trimester explant and to prolong cell proliferation [13]. Our SP cells from primary vCTB maintained the SP morphology in HSM containing FGF2 for at least 2 weeks after SP isolation. We did not check the later stage, but FGF2 is also a candidate mitogen for vCTB primary culture.

We also discovered that IL7R and IL1R2 are novel markers of SP cells derived from both HTR-8/SVneo and primary vCTB. Most of the SP cells expressed IL7R and IL1R2, but in contrast, NSP cells failed to express IL7R and IL1R2. It is known that IL7R is expressed on dendritic cells and monocytes, and activates multiple pathways that regulate lymphocyte survival, glucose uptake, proliferation and differentiation. IL7R signal in particular plays an essential role in T and B cell development and homeostasis [65], [66]. Although a previous study reported that IL7R was expressed in both vCTB and STB at an early stage of pregnancy its expression was rather weak [67]. The function of IL7R in trophoblast differentiation remains unknown. The other marker, IL1R2, was reported to antagonize IL1 activity by acting as a decoy target for IL1 in polymorphonuclear cells, a specific type of leukocyte [68]. IL1R2 expression has not been reported in placenta. Our *in vitro* data suggested that only a small proportion of vCTB cells expressed IL7R and IL1R2 and that they lost IL7R and IL1R2 expression as they differentiated into STB or EVT cells (Figure 6). Further investigation should reveal the function of IL7R and IL1R2 in the mechanism of human trophoblast differentiation.

Isolation of human TS cells is necessary to investigate the early trophoblast cell lineages with self-renewing properties and the capability to differentiate into all trophoblast cell types of the mature placenta. The pathology of pregnancy-associated complication is believed to be based on abnormal trophoblast differentiation, defects in trophoblast invasion and spiral artery remodeling. To understand the pathology, *in vitro* model using human TS cells will provide tremendous benefits. Furthermore, human TS cells may lead us to a new approach for treating patients with placental dysfunction with TS cell transfer. Our study provides new insights into the characteristics of human TS/ progenitor cells. This study also reveals several key factors that are practical and available markers for TS/ progenitor cell isolation, and which might be essential for the maintenance of TS/ progenitor cells. These new insights should help us to understand human TS cell biology and develop novel therapeutic technologies for placental disorders.

## Materials and Methods

### Cell culture

The HTR-8/SVneo trophoblast cell line was established elsewhere [68]. The parent HTR-8/SVneo cells, SP and NSP cells, and cells sorted by magnetic cell sorting (MACS) were cultured in either of the media shown in Table 1. Cells were cultured in 95 % air, and 5 % CO_2_ humidified atmosphere at 37°C. TCL-1 cells were maintained in RPMI1640 medium supplemented 10%FCS, 1% penicillin and streptomycin. Mouse embryonic fibroblasts (MEFs) were obtained from CD1 strain mouse fetuses at 16.5 dpc. After removal of the head, extremities and internal organs, fetal tissues were minced and trypsinized. For propagation, MEFs were cultured in DMEM supplemented with 10% FCS, 1% penicillin and streptomycin. MEFs were treated with 10 µg/ml mitomycin for 2 hours and were used as feeder cells or for preparation of conditioned medium.

### Preparation of human chorionic villi

Chorionic villous tissues from patients who voluntarily chose to terminate pregnancy during the first trimester (between 6^th^ and 9^th^ week of gestation) were obtained. These tissue samples were collected under the patients' informed consent and this study was conducted after reviewed and approved by Ethical Review Board of Kyushu University. The procedure was explained and written informed consent was obtained from all the patients. vCTB cells were isolated from the tissue samples as described elsewhere [69]. In brief, the villous tissues were minced finely and dissociated in Hanks' balanced salt solution (HBSS) containing HEPES (25 mmol), DNase1 and collagenase (15 U/ml) (Sigma, St. Louis, USA) for 30 min at 37°C with agitation. The dispersed cells were separated by filtration through a wire sieve (40 µm diameter pores) and stored in the presence of 10% FCS. After percoll gradient centrifugation, cells were obtained on the order of 1×10^6^ mono-dispersed cells.

### Isolation of SP cells

To identify and isolate HTR-8/SVneo SP cells, the cells were dislodged from the culture dishes with trypsin and EDTA, washed and suspended at a concentration of 1×10^6^ cells/ml in RPMI1640 containing 2% FCS. The cells were then labeled with 2.5 µg/ml Hoechst 33342 dye (Molecular Probes, Eugene, OR) for 120 minutes at 37°C, either alone or in combination with 50 µmol/L verapamil (Sigma-Aldrich, St. Louis, MO). Finally, the cells were counterstained with 1 µg/ml propidium iodide to label dead cells. The cells were then passed through EPICS ALTRA HyPerSort (Beckman Coulter, Fullerton, CA) using dual wavelength analysis (blue, 424–444 nm; red, 675 nm) after excitation with 350 nm UV light. Propidium iodide-positive dead cells were excluded from the analysis. SP and NSP cells were separated and seeded in HSM or HBM with mouse embryonic fibroblast (MEF) feeder cells. SP and NSP cell samples for RNA and protein analysis were collected on the next day after plating. The cells were cultured for 2 to 4 weeks.

### Cell growth assay

Cells were plated in HSM. Cells were passaged every time they approached confluence during the 2 months of cultivation. Viable cells were counted for 2 months. Cell counting was performed using a hemocytometer.

### RNA extractions and real-time reverse transcription-polymerase chain reaction (RT-PCR)

Cultured cells were washed with ice-cold PBS. Cells were harvested in 1 ml of Isogen reagent (Nippon Gene, Tokyo, Japan) according to the manufacturer's instructions and total RNA was extracted. One microgram of each total RNA preparation was then reverse-transcribed using an Oligo (dT) primer and SuperScript™ II Reverse Transcriptase (Invitrogen, Carlsbad, CA). Real-time RT-PCR was next carried out in a total volume of 20 µL using Brilliant 2 Fast SYBR Green QPCR master mix (Stratagene, La Jolla, CA). All PCRs were carried out in triplicate. Relative expression levels were calculated using the ddCT method [70] after normalization to those of a housekeeping gene, GAPDH. Primer sequences were listed in Table S1.

### Immunocytochemistry

Immunocytochemistry was performed with modification of the method reported by Kishino *et al* [71]. The cells were seeded on coverslips. After incubation, cells were fixed with acetone for 10 min and permeabilized with phosphate-buffered saline (PBS) containing 0.5% Triton-X. After blocking with 4% bovine serum albumin for 60 min, cells were incubated with primary antibodies (IL7R (C-20), CGB (C-20), CD9 (H-110), VIM (vimentin) (V9), ENG (endoglin, also known as CD105) (P3D1) and MCAM (melanoma cell adhesion molecule, also known as CD146) (P1H12) from Santa Cruz Biotechnology, Santa Cruz, CA; IL1R2 (MAB263) and CXCR7 (MAB4227) from R&D Systems, Inc., NE Minneapolis; CK7 (M7018) and CSH1 (A0137) from DAKO, Tokyo, Japan; HLA-G (MEM-G/9), PHLDA2 (ab5964) and EOMES (23345) from Abcam, Cambridge, UK; ITGAV/ITGB3 (MAB1976) from Chemicon International Inc., Temecula, CA; HLA-A, B, C (W6/32) from eBioscience., San Diego, CA), overnight at 4°C, followed by incubation with secondary antibody (Alexa Fluor 546-labeled goat anti-rabbit IgG (A11035), and goat anti-mouse IgG (A11030), Alexa Fluor 488-labeled goat-anti-rabbit (A11043) from Invitrogen by Life Technologies; FITC-labeled goat-anti mouse IgG (231750-FITC) from Ana Spec, Inc., Fremont, CA; FITC-conjugate AffiniPure Rabbit anti-goat IgG from SEIKAGAKU Corporation, Tokyo, Japan), for 45 min at room temperature; nuclei were counterstained with Hoechst 33852. After washing three times, cells were mounted onto slide glasses with shielding buffer. Cells were analyzed using a BIOREVO BZ-9000 fluorescence microscope (Keyence, Osaka, Japan).

### Magnetic cell sorting (MACS)

To purify cells that expressed IL7R or IL1R2, cultured cytotrophoblasts were selected by positive selection, using magnetic microbeads coated with anti-IL7R or anti-IL1R2 antibodies (R&D Systems, Inc.). Briefly, cells were incubated with 10 µl of polyclonal anti-IL7R antibody and 10 µl of monoclonal anti-IL1R2 antibody (200 µg/ ml) followed by incubation with 10 µl of anti-rabbit or anti-mouse antibody-coated magnetic microbeads (Miltenyi Biotec, Auburn, CA).

### Protein extractions and Western blot

Subconfluent cells were lysed with ice-cold cell lysis buffer (20 mM Tris-HCl [pH 8.0], 1% Triton X-100, 10% glycerol, 137 mM NaCl, 1.5 M MgCl_2_, 1 mM EGTA, 50 mM NaF, 1 mM Na_3_VO_4_, 1 mM PMSF, 1 µg/ml leupeptin, 10 µg/ml aprotinin). Protein concentrations were determined using the Coomassie Protein Assay (Pierce, Rockford, IL, USA). For Western blotting, equal amounts of cell lysates were separated on 10−15% SDS-polyacrylamide gels and transferred onto polyvinylidene difluoride membranes. The membranes were blocked in TBST (10 mM Tris-HCl [pH 7.4], 150 mM NaCl and 0.05% Tween20) containing 5% nonfat dry milk, and washed in TBST. The blots were then incubated with diluted primary antibodies (IL7R (C-20), CGB (C-20) BMP4 (N-16), Id2 (C-20) and GAPDG (FL-335) from Santa Cruz Biotechnology, IL1R2 (MAB263) and CXCR7 (MAB4227) from R&D Systems, Inc.). After incubation with each primary antibody, blots were incubated with horseradish peroxidase-linked anti-mouse or anti-rabbit antibodies (Amersham Biosciences, Little Chalfont, Bucks, UK) and analyzed with the ECL Plus system (Amersham Biosciences).

### DNA microarray

DNA microarray analysis was performed using the Human Genome U133 Plus2.0 (KURABO, Osaka, Japan). In brief, total RNA was prepared with Isogen (Nippon Gene, Tokyo, Japan) from HTR8/ SVneo SP or NSP cells. Data analysis was performed with GeneChip Operating software ver1.4 (Affymetrix, Santa Clara, CA) to determine signal intensity of each spot and its local background on microarrays. Analyzed data were selected using Microarray Data Analysis Tool (KURABO). (http://www.ncbi.nlm.nih.gov/geo/query/acc.cgi?acc=GSE27909).

Global scaling was performed with a target intensity of 500 for each chip. The intensity index for every probe set was averaged within a group, control (NSP) and object (SP). This averaged intensity was used to calculate signal fold change. In order to reduce false positives, each probe set was assigned a call of ‘P’ (present) or ‘A’ (absent). Probe sets that were ‘A’ across all arrays were filtered out. We calculated net intensity by subtracting the mean intensity of all pixels within the local background area from the mean intensity of all pixels within the spot area. We set the cutoff points as 3- or 0.33-fold change. The software normalized biases in net intensity between fluorescent dye channels in a microarray by global normalization.

### Data analysis

Data are represented as the means ± standard deviation (SD) and were analyzed with Student's *t*-test. A value of *p*<0.05 was considered statistically significant.

## Supporting Information

Table S1 All sequences were selected from GenBank and used by real-time RT-PCR.
(XLS)
